# Integrated Assessment of Skeletal Muscle Quantity and Quality Is Associated with Survival in Patients with Oesophagogastric Malignancies: A Retrospective Cohort Study

**DOI:** 10.3390/cancers18121987

**Published:** 2026-06-18

**Authors:** Yannick Deswysen, Pierre Trefois, Etienne Danse, Alix Collard, Marc Van den Eynde, Nicolas Lanthier

**Affiliations:** 1 Service de Chirurgie et Transplantation Abdominale, Institut Roi Albert II, Cliniques Universitaires Saint-Luc, Université Catholique de Louvain (UCLouvain), 1200 Brussels, Belgium; 2 Laboratoire de Gastroentérologie et d’Hépatologie, Institut de Recherche Expérimentale et Clinique, Université Catholique de Louvain (UCLouvain), 1200 Brussels, Belgium; nicolas.lanthier@saintluc.uclouvain.be; 3 Service de Radiologie, Cliniques Universitaires Saint-Luc, Université Catholique de Louvain (UCLouvain), 1200 Brussels, Belgium; pierre.trefois@saintluc.uclouvain.be (P.T.); etienne.danse@saintluc.uclouvain.be (E.D.); 4 Cellule Statistique, Clinical Trial Center, Cliniques Universitaires Saint-Luc, Université Catholique de Louvain (UCLouvain), 1200 Brussels, Belgium; alix.collard@saintluc.uclouvain.be; 5 Service d’Oncologie Médicale, Institut Roi Albert II, Cliniques Universitaires Saint-Luc, Université Catholique de Louvain (UCLouvain), 1200 Brussels, Belgium; marc.vandeneynde@saintluc.uclouvain.be; 6 Service d’Hépato-Gastroentérologie, Cliniques Universitaires Saint-Luc, Université Catholique de Louvain (UCLouvain), 1200 Brussels, Belgium

**Keywords:** myopenia, myosteatosis, skeletal muscle density, oesophagogastric cancer, body composition, systemic inflammation, intramuscular fat

## Abstract

*Background:* Oesophagogastric cancer is associated with poor survival and major alterations in body composition. This study investigated the relationship between myopenia and myosteatosis in patients with oesophagogastric cancer and evaluated their prognostic significance and association with systemic inflammation. *Methods:* In this retrospective single-centre study, 161 consecutive patients diagnosed with oesophagogastric cancer between 2019 and 2023 underwent computed tomography-based body-composition analysis at diagnosis. Skeletal muscle index (SMI) and skeletal muscle density (SMD) were measured at the third lumbar vertebra. Myopenia and myosteatosis were defined using Martin’s criteria. Associations with overall survival (OS) and progression-free survival (PFS), and systemic inflammation assessed using C-reactive protein (CRP), neutrophil-to-lymphocyte ratio (NLR), and platelet-to-lymphocyte ratio (PLR), were analysed using Kaplan–Meier and Cox regression analyses. *Results:* Among the 161 patients included (67.1% male; median age 66 years), myopenia and myosteatosis were highly prevalent (62.7% and 87.6%, respectively), despite a median body mass index (BMI) within the normal range. Lowest SMI tertile was significantly associated with poorer OS and PFS, whereas lowest SMD tertile showed markedly reduced OS. On multivariate analyses, lower SMD remained independently associated with OS and with PFS, whereas SMI lost significance after adjustment for clinical and inflammatory factors. The coexistence of myopenia and myosteatosis was associated with significantly worse survival outcomes. An exploratory continuous muscle score integrating muscle quantity and muscle quality was associated with OS (hazard ratio 1.28 per SD increase) and demonstrated moderate prognostic discrimination (concordance-index 0.63). *Conclusions*: Muscle quantity and quality represent complementary dimensions of cancer-associated muscle impairment in oesophagogastric cancer. Collectively, these results suggest that a continuous measure integrating both muscle quantity and muscle quality provides a more clinically informative assessment of muscle impairment than traditional binary definitions of myopenia and myosteatosis.

## Simple Summary

Cancers of the oesophagus and stomach carry a poor prognosis, partly because patients frequently lose muscle mass and muscle quality as their disease progresses. These changes are difficult to detect through routine clinical examination alone. In this study, we used body-composition measurements derived from standard staging scans to assess skeletal muscle quantity and quality simultaneously in 161 patients. We found that impaired muscle quality was a stronger and more independent predictor of survival than muscle quantity alone. Patients with both conditions had the worst outcomes. We further developed a simple continuous score combining the two measurements, which predicted survival more accurately than traditional yes/no classifications. These findings highlight the clinical value of routine scan-based muscle assessment and suggest that integrating both muscle dimensions into a single measure could help identify the most vulnerable patients and guide earlier nutritional and supportive care interventions.

## 1. Introduction

Oesophagogastric cancers, characterised by high morbidity and mortality, remain a major global health challenge [1]. Despite progress in surgical techniques and oncological treatments, overall survival remains poor, highlighting the need to identify additional prognostic factors, potentially leading to therapeutic target adjustments [2]. Among these, cancer-associated alterations in skeletal muscle have emerged as key determinants of patient outcomes.

Poor outcomes in oesophagogastric cancer are partly related to malnutrition, which is highly prevalent in this population and has major clinical consequences [3]. Nutritional impairment reduces tolerance to oncological treatments, increases postoperative complications, and contributes to immune dysfunction [4]. Importantly, malnutrition is frequently accompanied by cancer-associated muscle wasting, a central component of the cachexia spectrum [5].

Body composition has emerged as an important factor of treatment tolerance, postoperative morbidity, and survival. Alterations in skeletal muscle quantity and quality, such as sarcopenia and myosteatosis, have gained increasing attention in oncology. Sarcopenia is currently defined as a progressive skeletal muscle disorder combining reduced muscle mass and impaired muscle function or strength [6]. The term ‘myopenia’ is used instead throughout this manuscript to describe reduced skeletal muscle quantity, as the present study relied exclusively on Computed tomography (CT)-derived morphologic assessment without any functional evaluation [7]. Myosteatosis is characterised by an abnormal lipid infiltration within skeletal muscle tissue (and therefore consistent with a decline in quality) [8]. Together, these alterations reflect distinct, but complementary dimensions of cancer-associated muscle impairment. However, reported prevalence rates vary considerably across studies, reflecting the ongoing lack of consensus regarding diagnostic thresholds and imaging methodologies [9]. This lack of standardisation hinders cross-study comparisons and limits the integration of body-composition metrics into routine clinical practice.

CT-based analysis of body composition provides a reliable and widely available method to quantify skeletal muscle compartments [10]. It also allows the investigation of dynamic changes in body composition throughout the course of cancer and during oncological treatments. In parallel, systemic inflammation has emerged as a key determinant of cancer outcomes, and simple blood-based inflammatory markers such as C-reactive protein (CRP), neutrophil-to-lymphocyte ratio (NLR), and platelet-to-lymphocyte ratio (PLR) have been consistently associated with survival in gastrointestinal malignancies [4]. These markers reflect the complex interactions between tumour biology, host immune response, and metabolic alterations. In a recent review, we reported a high prevalence of myopenia and myosteatosis in patients with oesophagogastric cancer, together with potential associations with survival and systemic inflammation [9]. Despite the absence of international consensus on CT-derived thresholds for myopenia and myosteatosis, the cut-off values proposed by Martin et al. are currently among the most widely used in oncological research [11]. The diversity of body-composition phenotypes highlights the complex biological interplay between skeletal muscle, adipose tissue, nutritional status, and systemic inflammation. Adipose tissue in particular may also contribute to metabolic and inflammatory process, acting as an energy reservoir reflecting nutritional status and as a source of inflammatory mediators that may promote tumour progression in oesophagogastric cancer [12,13,14]. Importantly, these processes may be associated with variations in routinely available systemic inflammatory markers such as CRP, NLR, and PLR, which were specifically investigated in the present study.

Although myopenia and myosteatosis are most often investigated as separate binary entities, muscle quantity and muscle quality represent two complementary and closely interconnected dimensions of cancer-associated muscle impairment. Consequently, examining these alterations independently of each other only provides a partial understanding of cancer patients’ overall muscular decline. In this context, integrating muscle mass and muscle radiodensity into a single quantitative measure may better capture the severity of muscle impairment and provide a more comprehensive indicator of patient vulnerability. However, such composite muscle-based metrics remain largely under-investigated in oesophagogastric cancer.

The main objective of this retrospective study was to evaluate CT-derived muscle parameters in patients with oesophageal and gastric cancer and to investigate their associations with oncological outcomes, clinical characteristics, and systemic inflammatory markers. In addition, the prognostic value of an integrated muscle score combining muscle quantity and muscle quality was explored.

## 2. Materials and Methods

### 2.1. Patient Selection and Study Design

This single-centre retrospective study included all adult patients diagnosed with oesophageal, oesophagogastric junction, or gastric cancer between November 2019 and December 2023.

Eligibility criteria were: (1) age ≥ 18 years; (2) histologically confirmed diagnosis of primary cancer; (3) imaging at diagnosis evaluable for analysis; (4) having received oncological treatment (surgery, neoadjuvant therapy followed by surgery, or locoregional/systemic treatment); and (5) a minimum of 3 months’ follow-up post-diagnosis. All tumour stages were included, except superficial early-stage tumours treated by endoscopic resection alone.

All cases were systematically reviewed by a multidisciplinary tumour board to determine disease stage and define the optimal therapeutic approach.

Surgical procedures included oesophagectomy (2- or 3-field), total or partial gastrectomy, oesophagogastrectomy, and atypical resections. Neoadjuvant strategies followed institutional standards, including perioperative chemotherapy with FLOT (fluorouracil, leucovorin, oxaliplatin, docetaxel) or FOLFOX (folinic acid [leucovorin], fluorouracil [5-FU], and oxaliplatin) regimens, as well as CROSS chemoradiotherapy (paclitaxel, carboplatin, 41.4 Gy) for tumours staged ≥ T2. Patients presenting with metastatic disease received systemic treatment tailored for tumour biology, including chemotherapy, immunotherapy, or targeted therapies.

Clinical data collected included demographic data (age and sex), anthropometric measurements (height, weight, body mass index [BMI]), tumour-specific data (pathological data including T classification, lymph node metastasis, distant metastasis, histology), vital and oncological status, immuno-inflammatory blood markers (CRP), neutrophils, lymphocytes, platelets, NLR, PLR, biological nutritional parameters such as albumin, and morbi-mortality of surgical treatments (Esophagectomy Complications Consensus Group (ECCG) and the Dindo–Clavien classification) [15,16].

The primary endpoints were overall survival (OS), defined as the time from cancer diagnosis to death due to any cause, and progression-free survival (PFS), defined as the time from treatment initiation to documented disease progression or death. Patients without any event (i.e., without any progression, alive at the time of study completion, or lost to follow-up) were censored at the date of the last follow-up recorded in the medical file.

This study was approved by the Institutional Review Board (2023/31MAI/240—B403) and was conducted in accordance with the principles of the Declaration of Helsinki. Given the retrospective design of the study, the requirement for informed consent was waived.

### 2.2. CT-Based Body-Composition Analysis

Skeletal muscle mass was assessed by a non-contrast abdominal CT scan or a non-contrast CT component from positron emission tomography (PET-CT), performed for standard cancer care at diagnosis. CT-derived body-composition analysis was used to quantify skeletal muscle quantity and muscle quality. A single transverse CT image at the level of the third lumbar vertebra (L3), a validated anatomical landmark for whole-body skeletal muscle assessment, was analysed for each patient and tissue area estimated, using previously described Hounsfield unit (HU) thresholds and quantified by Slice-o-matic software version 4.3 (Tomovision, Montreal, QC, Canada) [17,18]. The most common and accepted HU range for skeletal muscle tissue is −29 to +150 HU, −190 to −30 HU for subcutaneous fat tissue, and −150 to −50 HU for visceral fat tissue. All non-contrast CT-scan images were analysed by a single trained observer who was blind to patient’s status [18].

As proposed by Martin et al., low muscularity, called myopenia, is defined by a skeletal muscle index (SMI) < 43 cm^2^/m^2^ for patients who were underweight or had a healthy BMI (BMI up to 24.9 kg/m^2^) or <53 cm^2^/m^2^ for overweight and obese patients (BMI 25 to 30 kg/m^2^) for men and <41 cm^2^/m^2^ for women [19].

Skeletal muscle density (SMD) measurement was based on the mean muscle radiation attenuation for the entire muscle area in HU based on a non-contrast CT-scan image [18]. Myosteatosis was defined using BMI specific skeletal muscle attenuation thresholds of <41 HU for patients who were underweight or had a healthy BMI (BMI ≤ 24.9 kg/m^2^) and <33 HU for overweight and obese patients (BMI ≥ 25 kg/m^2^) [11].

### 2.3. Statistical Analysis

Normality of continuous variables was assessed using the Shapiro–Wilk test. Between-group comparisons used Student’s *t*-test or Mann–Whitney U test as appropriate for continuous variables, and χ^2^ or Fisher’s exact tests for categorical variables. Correlations between body-composition metrics and biological or clinical variables were assessed using Pearson’s correlation coefficient.

For survival analyses, skeletal muscle index (SMI) and skeletal muscle density (SMD) were analysed both as continuous variables and as categorical variables based on cohort-specific tertiles (lowest tertile versus the two upper tertiles). Cox proportional hazard models were fitted using tertile-based categorical variables to improve clinical interpretability and facilitate translation into clinically applicable risk categories.

Survival probabilities (OS and PFS) were estimated using Kaplan–Meier curves and compared with log-rank tests. Hazard ratios (HRs) and 95% confidence intervals (CIs) were calculated using Cox proportional hazard models. Multivariate Cox regression models were constructed using a backward stepwise elimination approach. Variables significant on univariate analyses (*p* < 0.05) or deemed clinically relevant a priori, including age, BMI, metastatic status, and inflammatory markers, were included in multivariate models while taking into account the limited number of events in order to reduce the risk of model overfitting. Potential collinearity between markers was considered during multivariate model construction. Formal multicollinearity diagnostics were additionally performed for continuous variables included in the models. All variance inflation factors (VIFs) were below 2, indicating that multicollinearity did not appear to represent a significant issue in this sample. Proportional hazard assumptions were verified for all Cox proportional hazard models, including tertile-based analyses, using standard diagnostic methods.

Because the cohort included different tumour locations, histological subtypes, and disease stages, additional exploratory subgroup analyses using univariate and multivariate Cox regression models were performed by incorporating sex, tumour location (oesophagus/oesophagogastric junction versus stomach), and histological subtype (adenocarcinoma and squamous cell carcinoma) into the initial survival models.

The prognostic performance of the continuous muscle score was evaluated using Harrell’s concordance index (C-index). Internal validation of prognostic models was performed via bootstrap resampling (500 iterations), and optimism-corrected C-indices were derived. All analyses were conducted using SAS version 9.4 (SAS Institute Inc., Cary, NC, USA) and Stata software (version 12; StataCorp LP, College Station, TX, USA).

## 3. Results

### 3.1. Cohort Characteristics

A total of 161 patients with oesophageal, oesophagogastric junction, or gastric cancer were included in the study and underwent CT-based body-composition assessment at diagnosis. The median age was 66 years (range 23.0–88.0), and two-thirds were male (67.1%). The median BMI was 24.8 kg/m^2^ (15.1–41.7) (Table 1).

Tumours were located mostly in the oesophagus (49.1%), the stomach (27.3%), and the oesophagogastric junction (23.6%) (Table 1). Adenocarcinoma represented the most frequent histological subtype (64.6%), followed by squamous cell carcinoma (28.0%). Other rare histological subtypes included gastrointestinal stromal tumours, neuroendocrine tumours, mixed adenoneuroendocrine carcinomas, and plasmacytoma. They represented only a small minority of the cohort, and were retained to preserve the real-world nature of the study population. At diagnosis, 25.0% of patients presented metastatic disease. Most patients presented locally advanced tumours with frequent nodal involvement (Table 1).

### 3.2. Surgical Outcomes

Among the 161 patients included in the study, surgery with curative intent was performed in 79 patients (49.1%) (Supplementary Table S3). Neoadjuvant therapy was administered in 59 of the 79 surgical patients (74.7%), almost equally divided between chemotherapy (49.2%) and chemoradiotherapy (50.8%). Minimally invasive approaches were used in 74.7% of cases. The most common procedures were two-field oesophagectomy (46.8%), total gastrectomy (16.5%), and three-field oesophagectomy (12.7%). R0 resection was achieved in 98.7% of cases. Postoperative complications occurred in 40.5% of patients, with anastomotic leak being the most frequent complication (13.9%). Severe complications (Clavien–Dindo grade ≥ IIIa) occurred in 17.7%, while 30-day and 90-day mortality rates were 2.5% and 3.8%, respectively (Supplementary Table S3).

### 3.3. Body Composition Based on Martin’s Cut-Offs

Body-composition measurements were available for all 161 patients (Table 2). Median SMI was 44.4 cm^2^/m^2^ and median SMD 23.9 HU. According to Martin’s criteria, myopenia and myosteatosis were highly prevalent, affecting 62.7% and 87.6% of the patient population, respectively. The particularly high prevalence of myosteatosis observed in this cohort may reflect the limited applicability of currently available CT-derived thresholds in oesophagogastric cancer populations. The prevalence of myopenia was higher in women than in men (75.5% vs. 56.5%; *p* = 0.02), while myosteatosis was common in both sexes (92.5% vs. 85.2%; *p* = 0.18).

**Figure 1:**
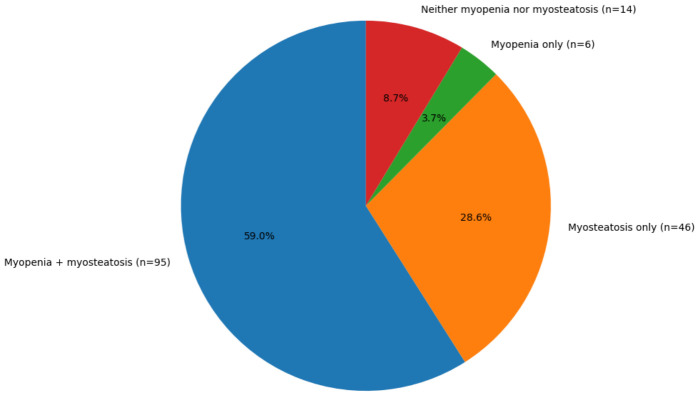
Distribution of muscle phenotypes (Martin’s criteria).

Baseline muscle phenotypes were distributed as follows: concomitant myopenia and myosteatosis (n = 95, 59.0%), isolated myosteatosis (n = 46, 28.6%), isolated myopenia (n = 6, 3.7%), and neither condition (n = 14, 8.7%) (Figure 1).

Notably, patients with myosteatosis also exhibited lower SMI compared with those without myosteatosis (42.3 vs. 52.6 cm^2^/m^2^, *p* < 0.0001), highlighting a substantial overlap between impaired muscle quality and reduced muscle quantity.

### 3.4. Body Composition According to Tertiles of Muscle Area and Density

Given the particularly high prevalence of myopenia and especially myosteatosis using Martin’s criteria in this cohort and the absence of universally accepted CT-derived thresholds for oesophagogastric cancer, skeletal muscle index (SMI) and skeletal muscle density (SMD) were analysed both as continuous variables and as categorical variables using cohort-specific tertiles. The lowest tertile was used as the reference category to explore potential gradient effects across increasing levels of muscle mass and muscle density.

When stratified by SMD tertiles, median SMI increased from 40.2 to 49.1 cm^2^/m^2^ from the lowest to the highest tertile, and the prevalence of myopenia decreased from 81.5% to 42.6% (Table 3). Myosteatosis was observed in 100% of patients in the two lowest SMD tertiles and in 63.0% in the highest tertile (Table 3). Patients in the lowest SMD tertile, compared with those in the two upper tertiles, were older and exhibited higher levels of systemic inflammation, including elevated CRP, consistent with a more pronounced catabolic and inflammatory phenotype (Supplementary Table S4). They also had lower SMI and a higher prevalence of myopenia compared with those in the upper tertiles (Table 3 and Supplementary Table S4). In contrast, no significant differences were observed for BMI or metastatic status across SMD tertiles (Supplementary Table S4).

Across SMI tertiles, median SMD increased from 20.4 to 29.0 HU, while the prevalence of myosteatosis decreased from 96.2% to 71.7% (Table 4). Accordingly, patients in the lowest SMI tertile had significantly lower BMI and SMD compared with those in the upper tertiles and exhibited a 100% prevalence of myopenia (Table 4 and Supplementary Table S5).

These findings highlight a strong interrelationship between skeletal muscle quantity and muscle quality, with lower muscle density frequently coexisting with reduced muscle mass.

### 3.5. Survival Analysis

To explore potential gradient effects, SMI and SMD were analysed as tertile-based categorical variables for Kaplan–Meier survival analyses and for Cox regression models assessing low- versus higher-tertile groups.

❖ *Survival according to SMI*

**Figure 2:**
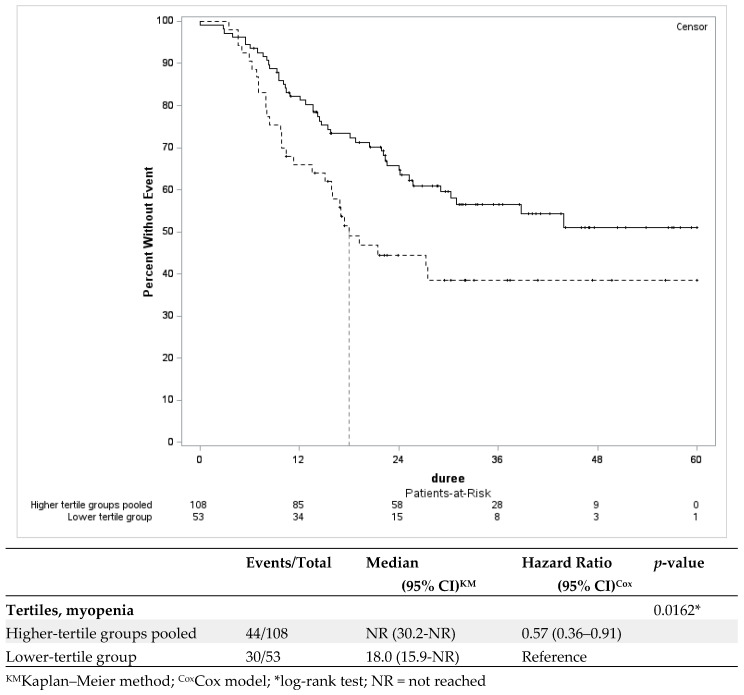
Kaplan–Meier survival curves of OS according to SMI tertile group. Dashed line: lowest tertile; solid line: higher tertiles.

**Figure 3:**
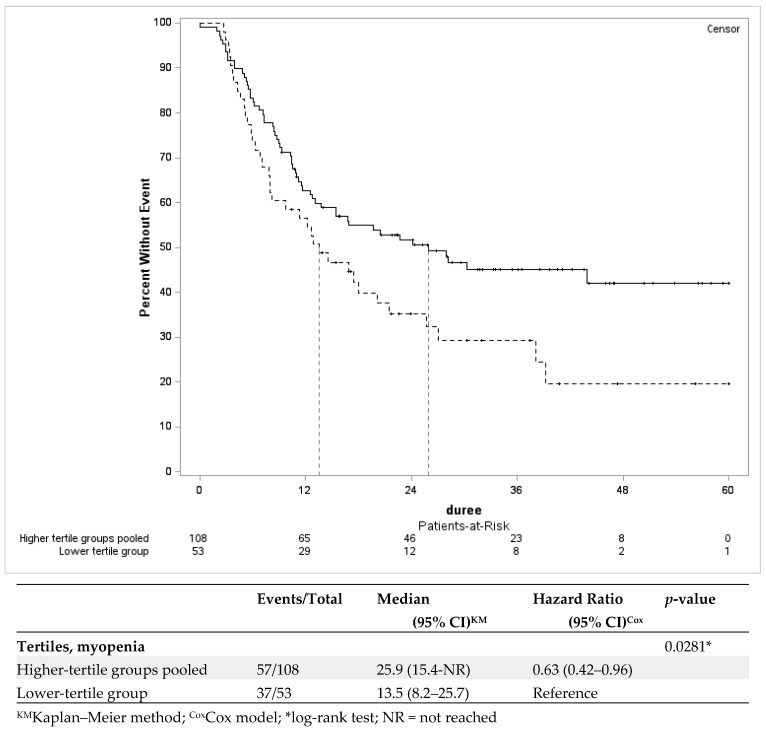
Kaplan–Meier survival curves of PFS according to SMI tertile group. Dashed line: lowest tertile; solid line: higher tertiles.

When stratified into tertiles, skeletal muscle mass was significantly associated with survival outcomes. Patients in the lowest SMI tertile experienced significantly poorer OS compared with those in the two upper tertiles combined (median OS: 18.0 months vs. not reached; HR 0.57; *p* = 0.016). A similar pattern was observed for PFS, with significantly shorter median PFS in the lowest tertile (13.5 vs. 25.9 months; HR 0.63; *p* = 0.028) (Figure 2 and Figure 3).

On univariate Cox regression analyses, low SMI tertile was significantly associated with both OS and PFS (Table 5 and Table 6). However, after adjustment for relevant clinical and inflammatory covariates, low SMI tertile did not retain independent prognostic significance in multivariate models for either OS or PFS, suggesting that the prognostic effect of muscle quantity may partly reflect underlying disease burden or systemic inflammation (Table 5 and Table 6).

❖ *Survival according to SMD*

**Figure 4:**
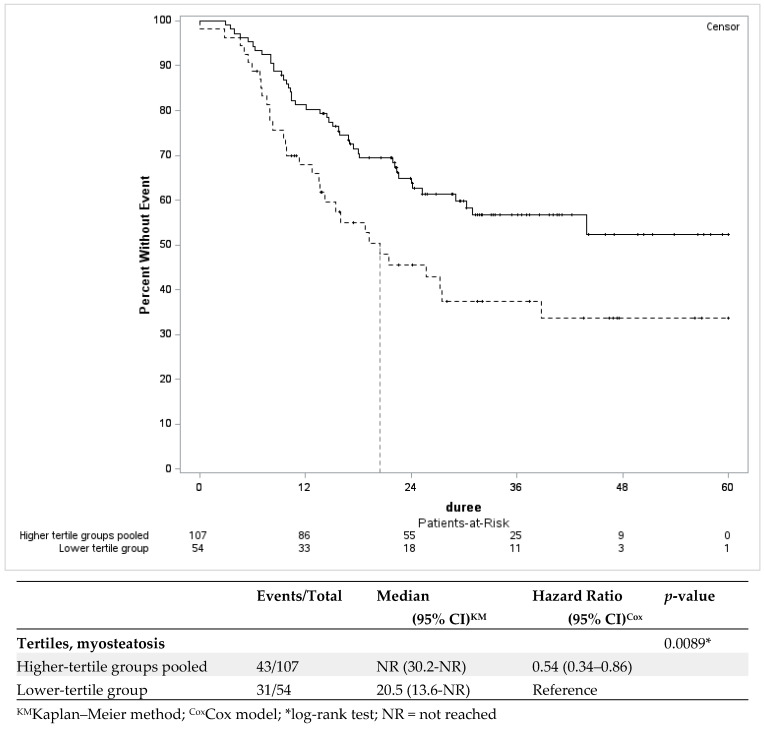
Kaplan-Meier survival curves of OS according to SMD tertile group. Dashed line: lowest tertile; solid line: higher tertiles.

**Figure 5:**
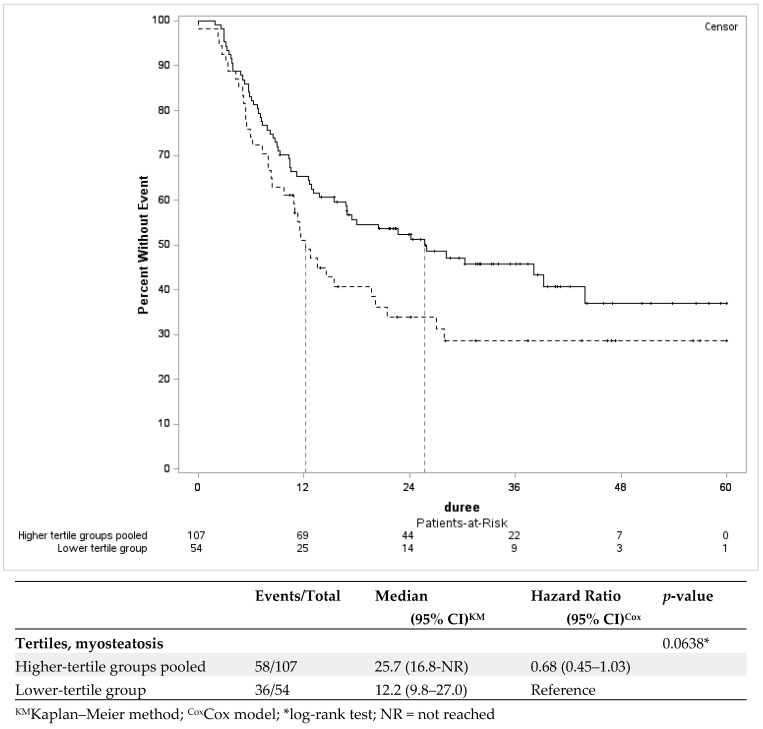
Kaplan–Meier survival curves of PFS according to SMD tertile group. Dashed line: lowest tertile; solid line: higher tertiles.

Skeletal muscle density demonstrated a stronger and more consistent prognostic impact than muscle mass. Patients in the lowest SMD tertile had significantly shorter OS compared with those in the middle and highest tertiles (median OS: 20.5 months vs. not reached; HR 0.54; *p* = 0.009) (Figure 4). Although the association with PFS did not reach conventional statistical significance, a trend toward poorer outcomes was observed (HR 0.68; *p* = 0.064) (Figure 5).

In multivariate Cox regression models adjusted for age, BMI, metastatic status, and systemic inflammatory markers, low SMD tertile remained independently associated with both OS and PFS (Table 5 and Table 6).

❖ *Survival according to other clinical and biological parameters*

On univariate analyses, BMI, metastatic disease, C-reactive protein (CRP), neutrophil-to-lymphocyte ratio (NLR), platelet-to-lymphocyte ratio (PLR), low SMI tertile, and low SMD tertile were all significantly associated with OS (Table 5). On multivariate analysis, metastatic disease and low-tertile SMD emerged to be the most significant predictors of mortality (HR 3.68, *p* < 0.001 and HR 0.46, *p* = 0.015 respectively) (Table 5).

For PFS, univariate analyses identified BMI, metastatic disease, CRP, NLR, PLR, low SMI tertile, and low SMD tertile as significant predictors. In multivariate models, metastatic disease (HR 3.23, *p* < 0.001) and PLR (*p* = 0.005) remained independently associated with shorter PFS. Similarly, low SMD tertile remained independently associated with shorter PFS on multivariate analyses (Table 6).

Overall, these findings suggest that muscle quality, as reflected by SMD, may represent a better prognostic marker for overall survival than muscle quantity in this population.

Additional exploratory univariate and multivariate analyses incorporating sex, tumour location, and histological subtype into the survival models are provided in Supplementary Tables S1 and S2. Overall, none of these additional clinicopathological variables materially modified the main associations observed between adverse muscle parameters and survival outcomes.

### 3.6. Towards a Combination of Myopenia and Myosteatosis

Four baseline muscle phenotypes were defined according to the presence or absence of myopenia and myosteatosis: combined myopenia–myosteatosis, isolated myopenia, isolated myosteatosis, and neither abnormality. Owing to the small subgroups, patients were dichotomised for survival analyses into those with the combined phenotype versus all other phenotypes.

**Figure 6:**
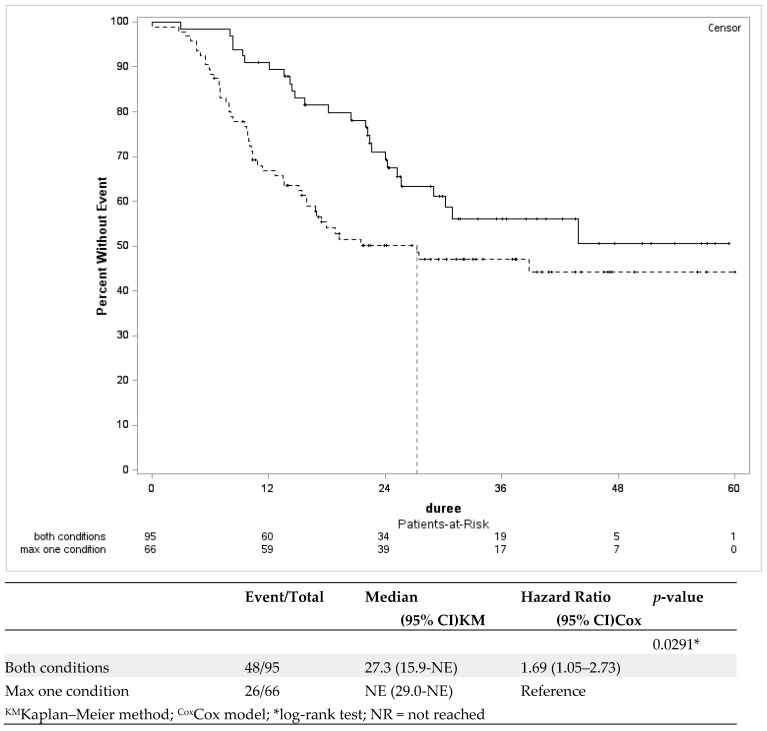
Kaplan–Meier survival curves of OS according to combined muscle phenotype. Dashed line: both conditions; solid line: max one condition.

**Figure 7:**
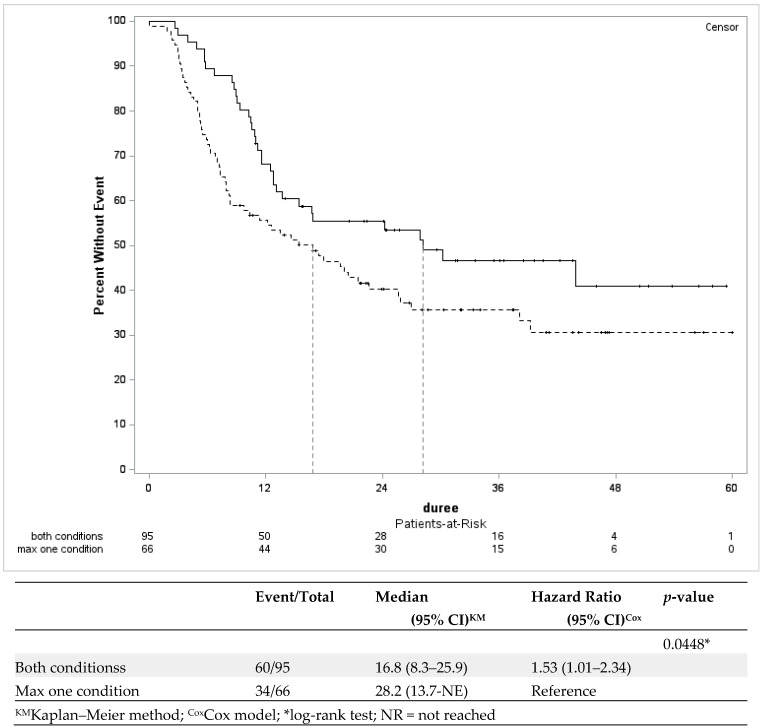
Kaplan–Meier survival curves of PFS according to combined muscle phenotype. Dashed line: both conditions; solid line: max one condition.

Given the substantial overlap between impaired muscle mass and muscle density—59.0% of patients exhibiting both myopenia and myosteatosis—survival was first assessed according to muscle phenotype. Patients presenting with the combined myopenia–myosteatosis phenotype experienced significantly poorer outcomes than those with one abnormality for both OS (HR 1.69, 95% CI 1.05–2.73; log-rank *p* = 0.029) and PFS (HR 1.53, 95% CI 1.01–2.34; log-rank *p* = 0.045) (Figure 6 and Figure 7). The persistence of an association with PFS for the combined phenotype may suggest that severe combined muscle impairment captures broader disease-related vulnerability than isolated muscle abnormalities alone. These findings suggest that the coexistence of reduced muscle mass and increased fat infiltration reflects a more advanced detrimental muscle phenotype.

To further explore outcome-driven thresholds, optimal cut-off values for SMI and SMD were identified within the study population using the Contal and O’Quigley outcome-oriented approach based on Mandrekar’s algorithm [20]. The optimal cut-offs were 44.44 cm^2^/m^2^ for SMI and 20.94 HU for SMD, corresponding to the values that maximised separation in overall survival (Supplementary Figure S1A,B).

Recognising the limitations of dichotomous definitions and the continuous nature of muscle impairment, we constructed a continuous composite muscle score integrating both muscle quantity and quality. SMI and SMD were standardised as cohort-based z-scores (z = [value − mean]/standard deviation) and combined as follows: Muscle score *=* −z(SMI) − z(SMD), with higher values indicating more severe muscle depletion and lower muscle radiodensity. Providing an explicit formula based on cohort-derived parameters facilitates reproducibility and future external validation. For practical application, the muscle score can be calculated as follows: −[(SMI − 45.15)/10.53] − [(SMD − 24.63)/9.58], where SMI is expressed in cm^2^/m^2^ and SMD in Hounsfield units (HU), and higher values indicate more severe muscle impairment. This continuous muscle score was strongly associated with overall survival. Each one-standard-deviation increase in the score was associated with a 28% increase in the risk of death (HR 1.28; *p* < 0.001). The score demonstrated a moderate, but robust discriminative performance (optimism-corrected C-index: 0.63) and showed clear separation of survival curves across tertiles, indicating its ability to capture progressive stages of muscle deterioration.

Collectively, these findings suggest that integrating muscle quantity and muscle quality into a continuous measure may provide a complementary and potentially clinically relevant assessment of muscle impairment compared with traditional binary definitions. However, this approach remains exploratory and requires further validation.

## 4. Discussion

The present study highlights the high prevalence of cancer-associated muscle impairment in patients with oesophagogastric cancer, characterised by both reduced skeletal muscle quantity and impaired muscle quality. Based on Martin’s criteria, myopenia was observed in 63% of patients and myosteatosis in 88%. These findings are consistent with previously published data reporting a high frequency of muscle mass depletion and muscle fat infiltration in upper gastrointestinal malignancies, reflecting the major metabolic alterations commonly associated with digestive cancers [5].

The present cohort intentionally included the spectrum of oesophageal, oesophagogastric junction, and gastric malignancies encountered in routine clinical practice. Although these tumours differ in terms of histology, oncological management, and prognosis, they frequently share common mechanisms contributing to nutritional deterioration and cancer-associated muscle impairment, including dysphagia, impaired oral intake, systemic inflammation, metabolic dysregulation, and cachexia. From this perspective, the present analyses were designed primarily to explore host-related vulnerability rather than tumour-specific oncological behaviour.

However, interpretation and comparison of prevalence estimates across studies remain challenging due to the lack of standardisation in diagnostic definitions [9] and imaging-derived thresholds. In the present cohort, the prevalence of myosteatosis reached 88% using Martin’s criteria, which appears particularly high and may suggest limited applicability of currently available cut-offs in patients with oesophagogastric cancer. To ensure methodological consistency, our primary analysis relied on the cut-offs proposed by Martin et al., which allow BMI- and sex-adjusted classification of both myopenia and myosteatosis and are widely used in oncological populations [11]. Nevertheless, thresholds used to define low muscle mass and low muscle attenuation vary substantially across studies depending on patient characteristics, imaging protocols, and analytical methodology, hence limiting the comparability of published results. This restriction further justified the complementary use of cohort-specific tertiles and continuous analyses in the present study. Thus, SMI and SMD were additionally categorised into cohort-based tertiles, primarily to explore potential dose–response relationships and facilitate graphical comparisons.

Stratification by muscle attenuation tertiles confirmed a close relationship between muscle quantity and muscle quality. Importantly, SMI-based stratification confirmed that myosteatosis remained frequent even among patients with preserved muscle mass, indicating that impaired muscle quality may occur independently of overt muscle depletion. These findings support the view that muscle mass assessment alone may underestimate the extent of muscle impairment in patients with oesophagogastric cancer.

From a prognostic perspective, low baseline SMI was associated with poorer OS and PFS in unadjusted analyses, while lower SMD showed a consistent association with worse OS and PFS. On multivariate analyses including factors traditionally associated with survival (metastatic status, systematic inflammatory markers), low SMD tertile remained independently associated with both OS and PFS after adjustment for clinical, inflammatory, and nutritional covariates. This suggests that muscle quality may represent an important prognostic determinant in this population and may better reflect the metabolic and inflammatory alterations associated with cancer-related muscle wasting. This association persisted despite adjustment for age, which is known to influence skeletal muscle radiodensity [21]. Importantly, when muscle phenotypes were examined together, the concomitant myopenia–myosteatosis phenotype identified a subgroup with significantly poorer overall survival compared with patients presenting with at most one alteration. This differential effect between OS and PFS suggests that muscle quality primarily reflects patient-related vulnerability rather than tumour aggressiveness, as tumour progression is mainly driven by tumour biology, whereas overall survival integrates both tumour characteristics and host-related factors [4,22]. The fact that the association between a low SMD and progression-free survival (PFS) remains significant after multivariate adjustment further suggests that part of its prognostic effect may be mediated by systemic inflammation and disease burden, as well as by tumour progression itself [4,5]. A growing body of literature has explored the combined impact of reduced muscle mass and myosteatosis on cancer outcomes. Although many studies use the term ‘sarcopenia’, most rely predominantly on CT-derived morphologic assessment without systematic functional evaluation. Studies in colorectal, biliary tract, liver, head and neck, prostate and resectable oesophageal cancers [23,24,25,26,27,28] have suggested that the coexistence of myopenia and myosteatosis identifies patients with particularly poor outcomes. Our findings are consistent with this literature, but extend it by focusing on a dedicated oesophagogastric cohort and by showing that muscle quality, inflammatory status, and the combined myopenia–myosteatosis phenotype provide complementary prognostic information [9,14,29].

The combined phenotype likely reflects a more advanced stage of muscle impairment, integrating both reduced muscle reserve and qualitative dysfunction, which may explain its stronger prognostic impact compared with isolated abnormalities. This observation is consistent with growing evidence that cancer-associated muscle alterations reflect not only nutritional depletion but also tumour-driven metabolic and inflammatory processes.

In our cohort, inflammatory markers were frequently elevated and significantly associated with outcomes on univariate analyses. CRP and PLR remained independently associated with overall survival in multivariate models, while metastatic status and PLR emerged as the dominant prognostic factors for progression-free survival. Systemic inflammation is a well-established adverse prognostic factor in gastrointestinal malignancies [4,22], and elevated CRP, NLR, PLR, and composite indices such as the modified Glasgow Prognostic Score have consistently been associated with poorer survival outcomes in gastric and oesophageal cancers [4,30,31,32]. These markers likely reflect complex interactions between tumour biology, host immunity, and metabolic alterations. Given the potential collinearity between CRP, NLR, and PLR, these findings should be interpreted with caution, as they may reflect overlapping biological processes related to host response and immune dysregulation [33,34]. Overall, our findings support the concept that muscle quality and systemic inflammation are closely interconnected, with myosteatosis potentially representing a downstream manifestation of chronic inflammatory and metabolic dysregulation in cancer patients.

Recent studies have also highlighted the potential prognostic relevance of the interaction between nutritional biomarkers and muscle quality, particularly through the emerging concept of the albumin–myosteatosis gauge [35]. Hypoalbuminemia and myosteatosis may represent complementary manifestations of cancer-associated metabolic impairment, integrating systemic inflammation, nutritional depletion, altered protein synthesis, and skeletal muscle lipid infiltration. In this context, myosteatosis should probably not be interpreted as an isolated radiological abnormality, but rather as part of a broader host-related metabolic and inflammatory phenotype associated with adverse oncological outcomes.

Building on these observations, we explored whether integrating muscle quantity and muscle quality into a single continuous muscle-based score could improve risk stratification. A composite score combining a standardised skeletal muscle index and skeletal muscle density was significantly associated with overall survival. This composite score assumes an equal contribution of muscle quantity and muscle quality, as no validated weighting scheme is currently available to differentially weight skeletal muscle index and skeletal muscle radiodensity in this population. Exploratory analyses supported the equal-weighting assumption used for score construction. Each one-standard-deviation increase in muscle score was associated with a 28% increase in the hazard of death, and the score showed a moderate performance for mortality prediction (bootstrap-corrected C-index 0.63). Internal validation using bootstrap resampling demonstrated minimal optimism, supporting the stability of this muscle-based score within the present cohort.

Clinically, these results support the potential utility of routine CT-based body-composition assessment using images acquired for staging and follow-up. CT-derived muscle assessment may help identify vulnerable patients who could benefit from earlier supportive interventions, such as structured prehabilitation, multimodal nutritional optimisation, resistance-based exercise programmes, and tailored symptom management during systemic therapy. Prehabilitation strategies that combine nutrition, physical activity, and anti-inflammatory approaches have shown promise in improving functional status and treatment tolerance in other oncological populations, and our findings suggest similar approaches may be beneficial in patients with oesophagogastric cancer [36].

The proposed muscle-based score may represent a simple and reproducible framework to summarise muscle impairment in daily practice and to facilitate prognostic stratification based on routinely available CT-derived measurements. The lack of independent association between muscle parameters and PFS further supports the hypothesis that tumour progression is primarily driven by tumour biology, whereas overall survival also reflects host-related vulnerability.

However, although the score demonstrated good internal validity, it should be considered exploratory, as it was developed and internally validated within a single retrospective cohort. External validation in independent cohorts—ideally within prospective study designs—is therefore required to confirm its generalisability, reproducibility, and clinical utility before routine clinical implementation.

This study has several strengths. It is based on a well-characterised real-world cohort of patients with oesophagogastric cancer, including a relatively large sample for a single-centre study. Body composition was assessed using validated CT-derived methods, and both established cut-offs (Martin et al.) and cohort-specific tertiles were used, allowing complementary analyses and exploration of potential dose–response relationships. In addition, the integration of routinely available systemic inflammatory markers (CRP, NLR, PLR) provides clinically relevant insight into the interplay between muscle alterations and inflammation.

Several limitations should also be acknowledged. The retrospective design and the biological heterogeneity in tumour locations, histological subtypes, and treatment strategies may have introduced bias and may partly influence survival outcomes independently of body-composition alterations. In addition, the requirement for a minimum follow-up duration may theoretically have introduced survivorship bias by excluding patients with very early deterioration, rapid progression, or early death. Similarly, the requirement for evaluable CT imaging may have introduced selection bias by excluding patients without adequate imaging quality or availability. Formal interobserver and intraobserver reproducibility analyses were not performed because of the retrospective design of the study, although all segmentations were conducted using a standardised methodology under radiological supervision. The inclusion of heterogeneous tumour locations, histological subtypes, metastatic status, and treatment intent may limit the biological homogeneity of the cohort. However, additional exploratory univariate and multivariate analyses incorporating sex, tumour location, and histological subtype into the survival models demonstrated globally consistent directions of association between adverse body-composition profiles and survival outcomes across tumour categories, although statistical power remained limited in several subgroup analyses. Accordingly, our results should not be interpreted as demonstrating equivalent tumour biology across oesophagogastric malignancies, but rather as highlighting the prognostic relevance of cancer-associated muscle impairment across upper gastrointestinal cancers. In addition, the absence of functional measures such as handgrip strength or gait speed precluded formal assessment of sarcopenia according to current consensus definitions, which require both muscle quantity and function. Subgroup analyses, particularly for patients presenting with a single abnormality (isolated myosteatosis or isolated myopenia), were limited by small samples. Furthermore, longitudinal evolution of body-composition parameters during treatment was not systematically assessed in the present study. Finally, variations in cachexia severity, nutritional support, and supportive care interventions during treatment may also have influenced body-composition changes and clinical outcomes.

## 5. Conclusions

In summary, CT-derived muscle abnormalities were highly prevalent in this cohort of upper gastrointestinal cancer patients and were associated with survival. Reduced muscle attenuation and the combined myopenia–myosteatosis phenotype identified patients with markedly poorer prognosis. A continuous muscle-based score integrating muscle quantity and quality was independently associated with overall survival and demonstrated moderate, but robust prognostic discrimination after internal validation. This score provides a pragmatic proof-of-concept approach for integrating muscle quantity and quality into a single continuous parameter, pending external validation.

## Figures and Tables

**Table 1:** Cohort description.

Variables	Median (IQR) or n (%)
**Age** (years)	66.0 (23.0–88.0)
**Sex**	
Male	108 (67.1%)
Female	53 (32.9%)
**BMI** (kg/m^2^)	24.8 (15.1–41.7)
**Localisation of tumour**	
Oesophagus	79 (49.1%)
Oesophagogastric junction	38 (23.6%)
Stomach	44 (27.3%)
**Histology**	
Adenocarcinoma	104 (64.6%)
Squamous cell carcinoma	45 (28.0%)
Other rare histologies	12 (7.4%)
**cTNM**	
T1	6 (3.7%)
T2	18 (11.2%)
T3	84 (52.2%)
T4	38 (23.6%)
Tx	15 (9.3%)
N0	7 (4.3%)
N1	30 (18.6%)
N2	79 (49.1%)
N3	36 (22.4%)
Nx	9 (5.6%)
M1	40 (25%)
**CRP** (mg/L)	3.7 (0.8–335.0)
**Platelets** (10^3^/µL)	262.0 (53.0–2434.0)
**Neutrophils** (per µL)	5.0 (1.4–15.7)
**Lymphocytes** (per µL)	1.6 (0.3–3.9)
**NLR**	3.8 (0.7–18.4)
**PLR**	172.0 (53.8–827.9)
**Serum albumin** (g/L)	42.0 (27.0–50.0)
**Surgery**	79 (49.1%)
**Neoadjuvant treatment**	59 (74.7%)
**Tumour recurrence**	69 (42.9%)

BMI, body mass index; CRP, C-reactive protein; NLR, ratio of neutrophils/lymphocytes; PLR, ratio of platelets/lymphocytes.

**Table 2:** Body-composition description.

Variables	Median (IQR) or n (%)		
	Total (n = 161)	Men (n = 108)	Women (n = 53)
SMI (cm^2^/m^2^)	44.4 (26.1–74.5)	44.5 (28.6–74.5)	44.0 (26.1–55.3)
SMD (HU)	23.9 (3.7–49.7)	24.0 (5.3–49.7)	23.9 (3.7–45.4)
Myopenia ^1^	101 (62.7%)	61 (56.5%)	40 (75.5%)
Myosteatosis ^1^	141 (87.6%)	92 (85.2%)	49 (92.5%)

SMI skeletal muscle index; SMD skeletal muscle density. ^1^ Myopenia and myosteatosis defined by Martin’s criteria.

**Table 3:** Body-composition description by tertile based on SMD.

Characteristic	All Patients, n = 161 Men 108 (67.1%) Women 53 (32.9%)	Low, n = 54 Men 30 (55.6%) Women 24 (44.4%)	Intermediate, n = 53 Men 38 (71.7%) Women 15 (28.3%)	High, n = 54 Men 40 (74.1%) Women 14 (25.9%)	*p*-Value
SMD (HU), median (IQR)					
All patients	23.9 (3.7–49.7)	15.1 (3.7–20.3)	23.9 (20.4–29.4)	34.0 (29.4–49.7)	Ref
Men	24.0 (26.6–49.7)				
Women	23.9 (3.7–45.4)				
SMI (cm^2^/m^2^), median (IQR)					
All patients	44.4 (26.1–74.5)	40.2 (26.1–63.7)	43.5 (27.6–67.4)	49.1 (28.6–74.5)	<0.001 *
Men	44.5 (28.6–74.5)				
Women	44.0 (26.1–55.3)				
Myopenia ^1^					
All patients	101 (62.7%)	44 (81.5%)	34 (64.2%)	23 (42.6%)	<0.001 †
Men	61 (56.5%)				
Women	40 (75.5%)				
Myosteatosis ^1^					
All patients	141 (87.6%)	53 (100.0%)	54 (100.0%)	34 (63.0%)	<0.001 †
Men	92 (85.2%)				
Women	49 (92.5%)				

SMI skeletal muscle index; SMD skeletal muscle density. ^1^ Myopenia and myosteatosis defined by Martin’s criteria. * Kruskal–Wallis test. † Chi-squared test.

**Table 4:** Body-composition description by tertile based on SMI.

Characteristic	All Patients, n = 161 Men 108 (67.1%) Women 53 (32.9%)	Low, n = 53 Men 16 (30.2%) Women 37 (69.8%)	Intermediate, n = 55 Men 43 (78.2%) Women 12 (21.8%)	High, n = 53 Men 49 (92.5%) Women 4 (7.5%)	*p*-Value
SMI (cm^2^/m^2^), median (IQR)					
All patients	44.4 (26.1–74.5)	34.8 (26.1–38.6)	44.4 (38.8–49.4)	55.3 (49.5–74.5)	Ref
Men	44.5 (28.6–74.5)				
Women	44.0 (26.1–55.3)				
SMD (HU), median (IQR)					
All patients	23.9 (3.7–49.7)	20.4 (3.7–41.8)	23.3 (6.2–49.7)	29.0 (5.3–45.4)	<0.001 *
Men	24.0 (26.6–49.7)				
Women	23.9 (3.7–45.4)				
Myopenia ^1^					
All patients	101 (62.7%)	53 (100.0%)	33 (60.0%)	15 (28.3%)	<0.001 †
Men	61 (56.5%)				
Women	40 (75.5%)				
Myosteatosis ^1^					
All patients	141 (87.6%)	51 (96.2%)	52 (94.5%)	38 (71.7%)	<0.001 †
Men	92 (85.2%)				
Women	49 (92.5%)				

SMI skeletal muscle index; SMD skeletal muscle density. ^1^ Myopenia and myosteatosis defined by Martin’s criteria. * Kruskal–Wallis test. † Chi-squared test.

**Table 5:** Univariate and multivariate analysis for overall survival (OS).

Variables	Univariate			Multivariate		
	HR	95% CI	*p*-Value	HR	95% CI	*p*-Value
Age	1.01	(0.986–1.029)	0.493	1.01	(0.988–1.038)	0.313
BMI	**0.94**	**(0.891–0.985)**	**0.01**	0.95	(0.894–1.010)	0.100
Metastasis	**3.92**	**(1.981–5.566)**	**<0.001**	**3.68**	**(1.846–6.565)**	**<0.001**
CRP	**1.01**	**(1.006–1.014)**	**<0.001**	**1.01**	**(1.001–1.011)**	**0.026**
NLR	**1.10**	**(1.021–1.183)**	**0.012**	0.95	(0.854–1.047)	0.283
PLR	**1.00**	**(1.001–1.004)**	**0.004**	**1.00**	**(1.001–1.005)**	**0.007**
Albumin	0.97	(0.944–1.001)	0.057	1.01	(0.970–1.049)	0.674
Low SMI tertile	**0.56**	**(0.339–0.935)**	**0.026**	0.82	(0.455–1.485)	0.516
Low SMD tertile	**0.53**	**(0.321–0.888)**	**0.016**	**0.46**	**(0.249–0.861)**	**0.015**

BMI body mass index; CRP, C-reactive protein; NLR ratio of neutrophils/lymphocytes; PLR ratio of platelets/lymphocytes; SMI: skeletal muscle index; SMD: skeletal muscle density. Values in bold are statistically significant. They are therefore distinguished from those that are not.

**Table 6:** Univariate and multivariate analysis for progression free survival (PFS).

Variables	Univariate			Multivariate		
	HR	95% CI	*p*-Value	HR	95% CI	*p*-Value
Age	1.00	(0.985–1.021)	0.761	1.00	(0.980–1.020)	0.958
BMI	**0.94**	**(0.903–0.986)**	**0.01**	0.96	(0.915–1.010)	0.121
Metastasis	**3.24**	**(2.022–5.179)**	**<0.001**	**3.38**	**(2.007–5.696)**	**<0.001**
CRP	**1.01**	**(1.005–1.012)**	**<0.001**	1.00	(0.998–1.007)	0.266
NLR	**1.18**	**(1.085–1.289)**	**<0.001**	1.05	(0.950–1.170)	0.319
PLR	**1.00**	**(1.001–1.004)**	**<0.001**	**1.00**	**(1.001–1.005)**	**0.002**
Albumin	0.98	(0.950–1.003)	0.079	1.01	(0.977–1.049)	0.504
Low SMI tertile	**0.62**	**(0.397–0.971)**	**0.037**	0.85	(0.519–1.396)	0.522
Low SMD tertile	**0.63**	**(0.399–0.988)**	**0.044**	**0.55**	**(0.320–0.939)**	**0.029**

BMI body mass index; CRP, C-reactive protein; NLR ratio of neutrophils/lymphocytes; PLR ratio of platelets/lymphocytes; SMI: skeletal muscle index; SMD: skeletal muscle density. Values in bold are statistically significant. They are therefore distinguished from those that are not.

## Data Availability

The data that support the findings of this study are not publicly available due to privacy and ethical restrictions, as they contain information that could compromise the privacy of research participants. Data may be available from the corresponding author upon reasonable request and subject to institutional approval.

## Supplementary Materials

The following supporting information can be downloaded at https://www.mdpi.com/article/10.3390/cancers18121987/s1. Supplementary Figure S1A. SMI best cut-off selected based on Contal and O’Quigley method using the Mandrekar approach: 44.44 cm^2^/m^2^. Supplementary Figure S1B. SMD best cut-off selected based on Contal and O’Quigley method using the Mandrekar approach: 20.94 HU. Supplementary Table S1. Univariate and multivariate analysis for overall survival (OS) including age, sex, localisation, and histology. Supplementary Table S2. Univariate and multivariate analysis for progression-free survival (PFS), including age, sex, localisation, and histology. Supplementary Table S3. Surgical and morbidity description. Supplementary Table S4. Baseline characteristics according to SMD tertile. Supplementary Table S5. Baseline characteristics according to SMI tertile.
