# Supplementary material for: Integrated Assessment of Skeletal Muscle Quantity and Quality Is Associated with Survival in Patients with Oesophagogastric Malignancies: A Retrospective Cohort Study
Source: Cancers (Basel). 2026 Jun 18;18(12):1987. doi: 10.3390/cancers18121987 (PMC13297275; doi:10.3390/cancers18121987)
Supplement: Supplementary file 1 [file cancers-18-01987-s001.zip › cancers-4345482-supplementary.pdf]

Supplementary

Supplementary Figure S1A. Optimal SMI cut-off identified using the Contal and O'Quigley method (Mandrekar approach): 44.44 cm²/m²

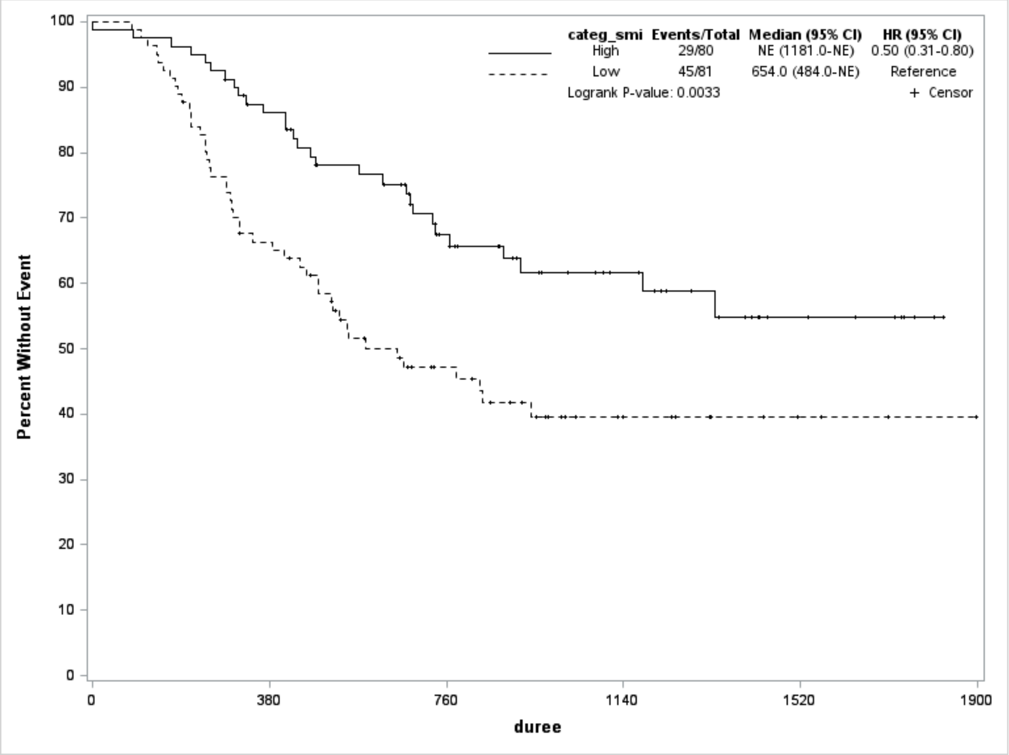

Supplementary Figure S1B. Optimal SMD cut-off identified using the Contal and O'Quigley method (Mandrekar approach): 20.94 HU

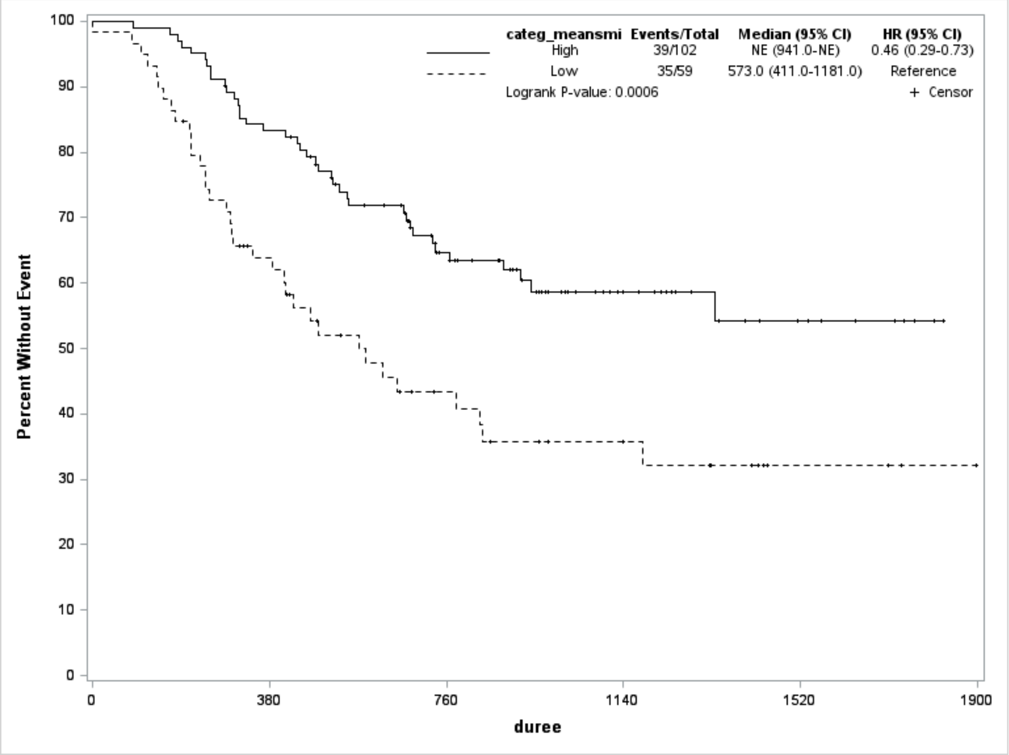

Supplementary Table S1. Univariate and multivariate analysis for overall survival (OS) including age, sex, localisation and histology

Variables	Univariate			Multivariate		
	HR	95% CI	P-value	HR	95% CI	P-value

Age	1.01	(0.986-1.029)	0.493	1.01	(0.982-1.035)	0.536
BMI	**0.94**	**(0.891-0.985)**	**0.01**	0.96	(0.892-1.022)	0.182
Metastasis	**3.32**	**(1.981-5.566)**	**<0.001**	**3.48**	**(1.846-6.565)**	**<0.001**
CRP	**1.01**	**(1.006-1.014)**	**<0.001**	1.01	(1.000-1.011)	0.038
NLR	**1.10**	**(1.021-1.183)**	**0.012**	0.93	(0.838-1.035)	0.185
PLR	**1.00**	**(1.001-1.004)**	**0.004**	**1.00**	**(1.001-1.005)**	**0.013**
Albumin	0.97	(0.944-1.001)	0.057	1.01	(0.968-1.047)	0.752
Low SMI tertile	**0.56**	**(0.339-0.935)**	**0.026**	0.58	(0.254-1.317)	0.192
Low SMD tertile	**0.53**	**(0.321-0.888)**	**0.016**	**0.42**	**(0.220-0.800)**	**0.008**
Genre	0.91	(0.539-1.527)	0.714	1.73	(0.753-3.977)	0.196
Histology (adenocarcinoma)	4.92	(0.676-35.810)	0.116	2.11	(0.274-16.288)	0.474
Histology (squamous cell carcinoma)	5.7	(0.766-42.47)	0.089	2.12	(0.25-18.036)	0.490
Localisation (stomach)	1.32	(0.763-2.287)	0.32	1.11	(0.545-2.270)	0.769

BMI body mass index ; CRP, C-reactive protein ; NLR ratio of neutrophils/lymphocytes ; PLR ratio of platelets / lymphocytes ; SMI: skeletal muscle index ; SMD : skeletal muscle density

Supplementary Table S2. Univariate and multivariate analysis for progression free survival (PFS) including age, sex, localisation and histology

Variables	Univariate			Multivariate		
	HR	95% CI	P-value	HR	95% CI	P-value
Age	1.00	(0.985-1.021)	0.761	1.00	(0.975-1.017)	0.718
BMI	**0.94**	**(0.903-0.986)**	**0.01**	0.97	(0.916-1.019)	0.209
Metastasis	**3.24**	**(2.022-5.179)**	**<0.001**	**3.17**	**(1.815-5.535)**	**<0.001**
CRP	**1.01**	**(1.005-1.012)**	**<0.001**	1.00	(0.998-1.007)	0.301
NLR	**1.18**	**(1.085-1.289)**	**<0.001**	1.05	(0.942-1.165)	0.395
PLR	**1.00**	**(1.001-1.004)**	**<0.001**	**1.00**	**(1.001-1.005)**	**0.004**
Albumin	0.98	(0.950-1.003)	0.079	1.01	(0.976-1.046)	0.542
Low SMI tertile	**0.62**	**(0.397-0.971)**	**0.037**	0.72	(0.371-1.402)	0.335
Low SMD tertile	**0.63**	**(0.399-0.988)**	**0.044**	**0.51**	**(0.295-0.895)**	**0.019**
Genre	0.92	(0.583-1.452)	0.721	1.41	(0.710-2.816)	0.345

Histology (adenocarcinoma)	3.12	(0.758-12.800)	0.115	1.66	(0.383-7.204)	0.498
Histology (squamous cell carcinoma)	3.78	(0.897-15.890)	0.070	1.95	(0.413-9.246)	0.398
Localisation (stomach)	1.29	(0.791-2.113)	0.305	1.20	(0.628-2.280)	0.585

BMI body mass index ; CRP, C-reactive protein ; NLR ratio of neutrophils/lymphocytes ; PLR ratio of platelets / lymphocytes ; SMI: skeletal muscle index ; SMD : skeletal muscle density

Supplementary Table S3. Surgical and morbidity description

Variables	Median (IQR) or n (%)
**Surgery**	79 (49.1%)
Primary surgery	16/79 (20.3%)
Neoadjuvant treatment	59/79 (74.7%)
Chemotherapy	29/59 (49.2%)
Radiochemotherapy	30/59 (50.8%)
Revision surgery	4/79 (5.1%)
**Minimal invasive surgery**	59/79 (74.7%)
**Type of surgery**	
2-fields oesophagectomy	37/79 (46.8%)
3-fields oesophagectomy	10/79 (12.7%)
Total oesophagogastrectomy	7/79 (8.9%)
Partial gastrectomy	7/79 (8.9%)
Total gastrectomy	13/79 (16.5%)
Atypical gastrectomy	3/79 (3.8%)
Other	2/79 (2.5%)
**R0 resection**	78/79 (98.7%)
**Postoperative mortality**	
Day 30	2/79 (2.5%)
Day 90	3/79 (3.8%)
**Surgical complications**	32/79 (40.5%)
Postoperative pneumonia	4/79 (5.1%)
Anastomotic leak	11/79 (13.9%)

Conduit necrosis	2/79 (2.5%)
Lesion of the recurrent nerve	1/79 (1.3%)
Severe complications (> IIIa Clavien-Dindo classification's grade)	14/79 (17.7%)

Supplementary Table S4. Baseline characteristics according to SMD tertiles

Variables	Low, n=54	Intermediate, n=53	High, n=54	P-value
Age, years	73.5 [63.2–79.0]	67.0 [61.0–74.0]	60.0 [53.2–65.0]	<0.001
Male sex, n (%)	30 (55.6)	38 (71.7)	40 (74.1)	0.084
BMI, kg/m²	24.9 [20.7–28.9]	25.1 [22.7–30.1]	24.2 [21.3–26.8]	0.666
Metastatic disease, n (%)	12 (22.6)	16 (30.2)	12 (22.2)	0.565
CRP, mg/L	4.9 [2.1–13.3]	5.8 [1.8–30.8]	1.5 [1.0–6.4]	<0.001
NLR	4.03 [2.34–4.98]	3.30 [2.60–4.21]	2.71 [2.16–4.10]	0.109
PLR	168.0 [126.3–246.4]	167.0 [133.3–220.1]	179.2 [115.1–234.8]	0.990
Albumin, g/L	41.0 [39.0–44.0]	42.0 [38.5–44.0]	42.0 [40.0–45.0]	0.317
SMI, cm²/m²	40.2 [26.1–63.7]	43.5 [27.6–67.4]	49.1 [28.6–74.5]	<0.001
SMD, HU	15.1 [3.7–20.3]	23.9 [20.4–29.4]	34.0 [29.4–49.7]	<0.001
Myopenia, n (%)	44 (81.5)	34 (64.2)	23 (42.6)	<0.001
Myosteatosis, n (%)	53 (100.0)	54 (100.0)	34 (63.0)	<0.001

BMI body mass index ; CRP, C-reactive protein ; NLR ratio of neutrophils/lymphocytes ; PLR ratio of platelets / lymphocytes ; SMI, skeletal muscle index ; SMD, skeletal muscle density
Continuous variables are expressed as median (interquartile range) and categorical variables as number (%). Comparisons between groups were performed using the Kruskal–Wallis test or χ^2 test as appropriate.

Supplementary Table S5. Baseline characteristics according to SMI tertiles

Variables	Low, n=53	Intermediate, n=55	High, n=53	P-value
Age, years	70.0 [60.0–77.0]	67.0 [60.0–72.0]	63.0 [55.0–69.0]	0.041
Male sex, n (%)	16 (30.2)	43 (78.2)	49 (92.5)	0.006
BMI, kg/m²	22.7 [20.0–26.0]	25.0 [22.5–28.3]	26.5 [23.3–29.8]	<0.001
Metastatic disease, n (%)	13 (24.5)	14 (25.5)	13 (24.5)	0.989
CRP, mg/L	4.7 [1.9–18.4]	3.7 [1.5–11.2]	2.0 [1.0–7.3]	0.073

NLR	3.85 [2.31–4.78]	3.14 [2.48–4.31]	2.91 [2.11–3.98]	0.242
PLR	170.2 [129.4–238.6]	169.1 [125.3–220.4]	174.6 [117.2–231.8]	0.964
Albumin, g/L	41.0 [38.0–43.0]	42.0 [39.0–44.0]	42.0 [40.0–45.0]	0.285
SMI, cm^2/m^2	34.8 [26.1–38.6]	44.4 [38.8–49.4]	55.3 [49.5–74.5]	<0.001
SMD, HU	20.4 [3.7–41.8]	23.3 [6.2–49.7]	29.0 [5.3–45.4]	0.002
Myopenia, n (%)	53 (100.0)	33 (60.0)	15 (28.3)	<0.001
Myosteatosis, n (%)	51 (96.2)	49 (94.5)	38 (71.7)	0.002

BMI body mass index ; CRP, C-reactive protein ; NLR ratio of neutrophils/lymphocytes ; PLR ratio of platelets / lymphocytes ; SMI, skeletal muscle index ; SMD, skeletal muscle density
Continuous variables are expressed as median (interquartile range) and categorical variables as number (%). Comparisons between groups were performed using the Kruskal–Wallis test or χ^2 test as appropriate.
